# Impact of Liver and Kidney Function on Vitamin D3 Metabolism in Female and Male Patients Undergoing Allogeneic Hematopoietic Stem-Cell Transplantation

**DOI:** 10.3390/ijms26072866

**Published:** 2025-03-21

**Authors:** Laura Weich, Christina Brummer, Sakhila Ghimire, Katrin Peter, Michael Althammer, Nathalie Babl, Florian Voll, Christina Bruss, Marcus Hoering, Stefan Wallner, Peter J. Siska, Ernst Holler, Wolfgang Herr, Heiko Bruns, Iris M. Heid, Klaus Stark, Marina Kreutz, Carina Matos

**Affiliations:** 1 Department of Internal Medicine III, Hematology and Medical Oncology, University Medical Center of Regensburg, 93053 Regensburg, Germany; weich.laura@t-online.com (L.W.); sakhila.ghimire@ukr.de (S.G.); katrin.1.peter@gmail.com (K.P.); michael.althammer@ukr.de (M.A.); nathalie.babl@ukr.de (N.B.); florian.voll@ukr.de (F.V.); christina.bruss@ukr.de (C.B.); ernst.holler@ukr.de (E.H.); wolfgang.herr@ukr.de (W.H.); marina.kreutz@ukr.de (M.K.); 2 Institute of Clinical Chemistry and Laboratory Medicine, University Hospital Regensburg, 93053 Regensburg, Germany; marcus.hoering@ukr.de (M.H.); stefan.wallner@ukr.de (S.W.); 3 Department of Medicine 5, Hematology and Oncology, Friedrich-Alexander-Universität Erlangen-Nürnberg and University Hospital Erlangen, 91054 Erlangen, Germany; heiko.bruns@uk-erlangen.de; 4 Department of Genetic Epidemiology, University of Regensburg, 93053 Regensburg, Germany; iris.heid@ukr.de (I.M.H.); klaus.stark@ukr.de (K.S.)

**Keywords:** vitamin D3, 25(OH)D3 2, 1,25(OH), HSCT, GvHD, bilirubin, eGFR

## Abstract

We previously described that elevated levels of the active vitamin D3 metabolite 1,25-dihydroxyvitamin D3 (1,25(OH)2D3) during the early phase of allogeneic hematopoietic stem-cell transplantation (HSCT) can predict one-year transplant-related mortality (1y-TRM). Given that the liver and kidneys are the primary organs responsible for the effective conversion of vitamin D3, we investigated whether liver and/or kidney function, inflammation, or patient sex might influence vitamin D3 metabolism and, consequently, patient outcomes during transplantation. We found that female patients exhibited higher levels of 1,25(OH)2D3 at the time of transplantation compared with male patients. However, 1,25(OH)2D3 levels were associated with 1y-TRM in both sexes. No correlation was found between liver-associated markers, such as bilirubin, or the inflammation marker C-reactive protein (CRP) and serum levels of vitamin D3 metabolites in either female or male patients. However, serum levels of 1,25(OH)2D3, but not 25(OH)D3 correlated with the creatinine-based estimated glomerular filtration rate (eGFR), indicating that 1,25(OH)2D3 levels are associated with kidney function in HSCT patients. However, a Cox regression analysis, adjusted for baseline risk factors, demonstrated that high peri-transplant levels of 1,25(OH)2D3 (measured from days −2 to 7) remained a significant predictor of patient survival, even when eGFR was taken into account (hazard ratio = 0.99; *p* = 0.004). These findings suggest that optimal serum levels of 1,25(OH)2D3 may not be achievable in some HSCT patients and that kidney function alone cannot explain why some patients fail to reach the optimal 1,25(OH)2D3 threshold. These data support the potential use of 1,25(OH)2D3 as a prophylactic agent, particularly in patients with pre-existing kidney disease.

## 1. Introduction

Vitamin D3 is a fat-soluble vitamin that is mainly synthetized in the skin from 7-dehydrocholesterol under UVB irradiation. Vitamin D3 binds to vitamin D-binding protein (DBP) and is transported to the liver where it is converted to 25(OH)D3, the major circulating form of vitamin D3. In the kidneys, 25(OH)D3 is further metabolized into the biologically active vitamin D3 metabolite 1,25(OH)2D3 through the enzymatic action of CYP27B1 [1,2,3]. The production of 1,25(OH)2D3 is tightly regulated through both negative and positive feedback mechanisms, primarily involving three key hormones: parathyroid hormone (PTH), fibroblast growth factor 23 (FGF23), and 1,25(OH)2D3 itself. In the kidney, the enzyme CYP27B1 is primarily stimulated by PTH, while FGF23 and 1,25(OH)2D3 inhibit it [4]. The levels of 1,25(OH)2D3 are also regulated by CYP24A1, the enzyme responsible for 1,25(OH)2D3 catabolism [4]. In immune cells, CYP27B1 is regulated—for example, by activation via IFNγ [5]. 1,25(OH)2D3 enters the cell and binds to the intracellular vitamin D3 receptor (VDR), a transcription factor, that, in turn, can regulate gene transcription in collaboration with its partner, the retinoid X receptor (RXR) [6].Although classically known for maintaining calcium homeostasis and bone mineralization, it is well acknowledged that vitamin D3 has other important roles in the human body. Among them, vitamin D3 regulates cell proliferation, immune-cell function, and hematopoietic-cell differentiation [7]. Effects of vitamin D3 metabolites on hematopoietic stem-cell transplantation (HSCT) have also been documented; however, most studies have focused on 25(OH)D3 and the results are inconsistent [8,9,10,11]. We previously showed that early serum levels of 1,25(OH)2D3, but not of its metabolic precursor 25(OH)D3, are predictive for survival after HSCT, as lower 1,25(OH)2D3 levels around HSCT (days −2 to 7) were associated with higher risk of 1-year treatment-related mortality (TRM) [12].

Allogeneic HSCT is a potentially curative therapy to treat hematopoietic malignancies. Patients receive conditioning chemotherapy or irradiation to eradicate malignant cells prior to the infusion of donor stem cells. Conditioning can lead to tissue damage and release of inflammatory cytokines. Inflammation may increase the plasma concentration of the C-reactive protein (CRP), a liver-derived acute-phase protein, which has been described as an independent risk factor for transplant-related complications 21 days after allogeneic HSCT [13]. Commonly, after allogeneic HSCT, liver damage leading to hyperbilirubinemia [14] occurs. Causes include endothelial complications such as hepatic veno-occlusive disease (VOD) or acute graft-versus-host disease (GvHD).

GvHD is a common complication that can arise following stem-cell infusion and engraftment. It typically presents as damage to the skin, as well as the lower and upper gastrointestinal tracts, and the liver [15]. Additionally, acute kidney injury may occur, which is usually characterized by a temporary decrease in estimated glomerular filtration rate (eGFR) based on serum creatinine levels [16,17,18]. This condition has been reported to affect up to half of patients undergoing allogeneic hematopoietic stem-cell transplantation (HSCT) during the initial post-transplant period [17]. Acute kidney injury is often linked to poorer overall survival rates and increased transplant-related mortality [19].

Additionally, sex differences are being acknowledged in disease manifestation. Chronic kidney disease affects more women than men; however, kidney function declines more rapidly in men than in women, likely due to testosterone and unhealthier lifestyles [20]. It is also known that men tend to have higher levels of bilirubin and creatinine compared with women [21,22,23]. Furthermore, there is an interaction between vitamin D3 and estrogen levels. Vitamin D3 enhances the bioavailability of estrogens, while estrogens may improve the efficacy of vitamin D3 absorption, transport systems, and vitamin D receptor (VDR) expression [24].

As both the liver and kidneys are essential organs for vitamin D3 metabolism, transplant-associated hepatic or renal toxicity may also impact this process. Therefore, we aimed to investigate whether fluctuations in vitamin D3 levels are related to changes in liver and/or kidney function. To achieve this, we monitored total levels of 25(OH)D3, 1,25(OH)2D3, bilirubin as a marker of liver function, and the creatinine-based estimated glomerular filtration rate (eGFR) as a marker of kidney function during inpatient stays and thereafter during routine outpatient visits. We conducted the analysis separately for women and men.

## 2. Results

### 2.1. Time Trend of 1,25(OH)2D3 Serum Levels in Association with Patient Sex

**Figure 1:**
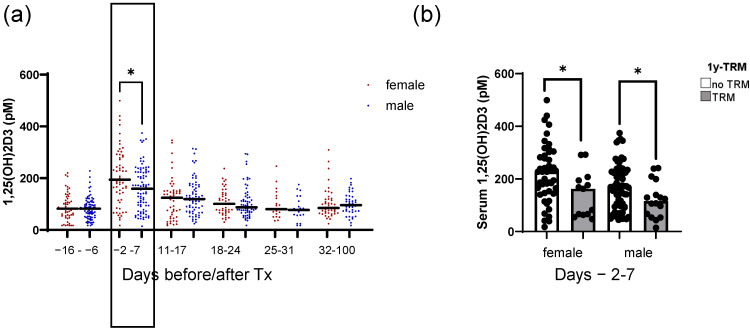
Time trend of 1,25-dihydroxyvitamin D3 serum levels in association with patient sex. Shown in (**a**) are the serum 1,25(OH)2D3 serum levels for female and male patients of the analyzed cohort, measured repeatedly at hospital admission (days −16 to −6); peri-transplant (days −2 to 7); during early weekly follow-up (days 11 to 17; 18 to 24; 25 to 31); and late follow-up (days 32 to 100 after HSCT). In (**b**), the 1,25(OH)2D3 serum levels are depicted for female and male patients with or without TRM. Median serum levels are depicted in bold. Statistical analysis was performed using the Mann–Whitney U test (* *p* ≤ 0.05).

We previously identified peri-transplant 1,25(OH)2D3 levels as being a means to predict first-year TRM [10]. In the present analysis, we evaluated the time trend of 1,25(OH)2D3 levels in relation to patient sex (women *n* = 59; men *n* = 82). We identified a significant difference in 1,25(OH)2D3 levels between females and males, with females showing a higher median level compared with males around transplantation time (days −2 to 7; 194 vs. 159 pM, respectively, *p* = 0.017; Figure 1a). However, in both females and males, higher 1,25(OH)2D3 levels were associated with lower 1-year transplant-related mortality (1y-TRM) (*p* = 0.035 and 0.036 for women and men, respectively; Figure 1b).

### 2.2. Impact of Sex on Peri-Transplant Liver and Kidney Parameters

**Figure 2:**
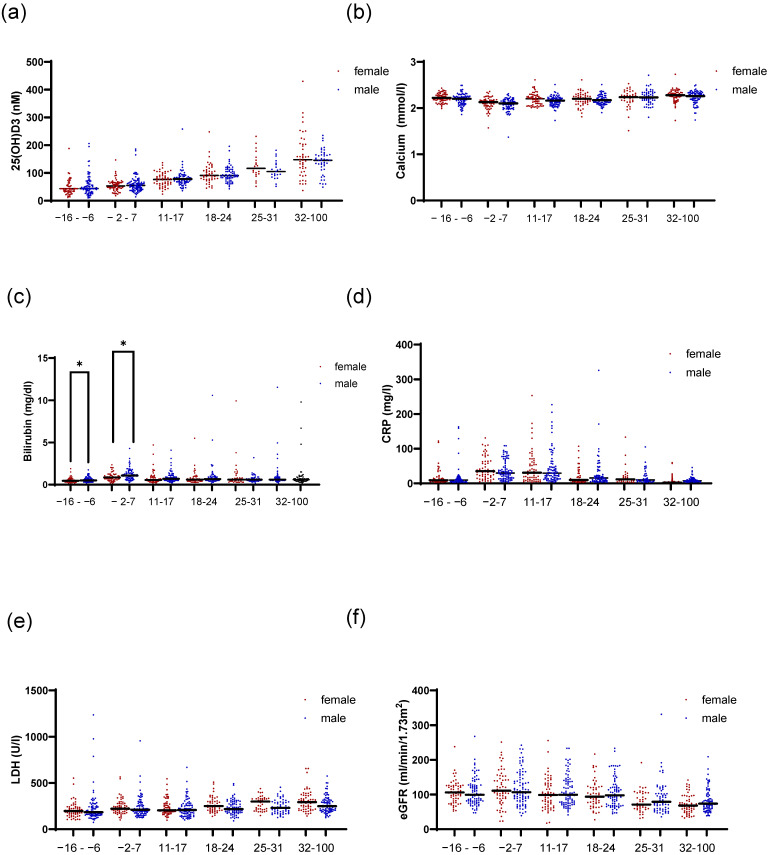
Time trend of clinical liver and kidney parameters in association with patient sex. Figure (**a**) shows the 25(OH)D3 serum levels for female and male patients over time in the indicated time intervals. In (**b**), calcium levels are depicted. In (**c**), the total amount of serum bilirubin is presented. In (**d**), CRP serum levels are depicted for female and male patients. In (**e**), LDH levels are shown and in (**f**), the eGFR values are shown. Statistical analysis was performed using the Mann–Whitney U test (* *p* ≤ 0.05).

As we observed differences in 1,25(OH)2D3 serum levels between female and male patients around the time of HSCT, we sought to investigate clinical parameters that might influence 1,25(OH)2D3 levels in these patients. Based on the importance of liver and kidney function for vitamin D metabolism, we analyzed 25(OH)D3, calcium, phosphate, bilirubin, CRP, LDH, and eGFR as factors that mirror the functionality of liver and kidney. No differences in the levels of 25(OH)D3 or calcium were detected between female and male patients (Figure 2a,b). We also analyzed calcium and phosphate in relation to our previously calculated optimal threshold for 1,25(OH)2D3 [12] and median 25(OH)D3 levels (Figures S1 and S2). We observed higher calcium in sera of females with higher 1,25(OH)2D3 only from day 18 onwards. Males with lower serum 1,25(OH)2D3 showed slight but significantly higher calcium levels only in the time point before transplantation. For phosphate, no differences were detected in females in relation to 25(OH)2D3 levels (Figure S2A). Additionally, we observed that during the time interval of days 18–24 after HSCT, female patients with higher 1,25(OH)2D3 had higher phosphate serum levels (Figure S2B). For male patients, we observed that at peri-transplant (days −2–7) and the time interval after (days 11–17), male patients with higher 25(OH)D3 had lower phosphate serum levels. No differences were observed in relation to 1,25(OH)2D3. As expected, male patients showed higher total bilirubin levels compared with females (Figure 2c). This was only significant during the time intervals before transplantation. The C-reactive protein (CRP) values were also evaluated as a readout of inflammation, and no difference between female and males was observed (Figure 2d). However, when we analyzed CRP levels in relation to the 1,25(OH)2D3 cutoff, for female and male patients separately (Figure S3), we observed that females with lower 1,25(OH)2D3 levels had higher CRP values, indicating that the reduced 1,25(OH)2D3 levels may have resulted from inflammation. Lactate dehydrogenase (LDH) can be used to check for tissue damage or injury. Although it is recognized that male patients may have slightly higher values compared with females, our analysis did not reveal significant changes (Figure 2e). When analyzing LDH levels in relation to 1,25(OH)2D3 levels, we observed that for females, on days 11–17 and 18–24 after transplantation, patients with lower 1,25(OH)2D3 had higher serum LDH (Figure S4A). No differences were detected for male patients (Figure S4C). Figure 2f shows the eGFRs in female and male patients, and no significant changes were observed between female and male patients during the analyzed time points.

### 2.3. Correlation of Bilirubin Levels with 25(OH)D3 and 1,25(OH)2D3 in Female and Male Patients

**Figure 3:**
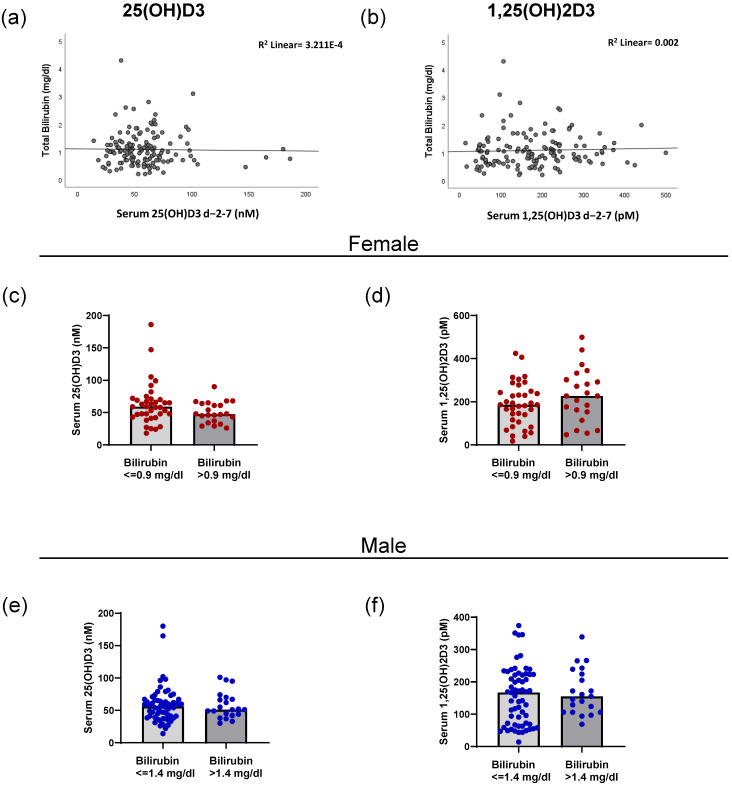
Correlation of bilirubin levels with 25-hydroxyvitamin D3 and 1,25-dihydroxyvitamin D3 in female and male patients. The correlation between total bilirubin levels and 25(OH)D3 (**a**) and 1,25(OH)2D3 (**b**) in serum for the peri-transplant time interval are shown. (**c**) The level of serum 25(OH)D3 in female patients above and below the sex-specific bilirubin cutoff of 0.9 mg/dL. (**d**) The amount of 1,25(OH)2D3 in the serum of female patients in relation to the bilirubin cutoff. (**e**) The amount of 25(OH)D3 in the serum of male patients in relation to the sex-specific cutoff of 1.4 mg/dL of bilirubin. (**f**) The serum 1,25(OH)2D3 levels of male patients in relation to the bilirubin cutoff.

The 25(OH)D3 metabolite is mainly produced in the liver by the action of CYP enzymes such as CYP2R1 [2]. To analyze whether possible malfunctions in the liver could be linked with vitamin D3 metabolite levels, we correlated total bilirubin levels with 25(OH)D3 and 1,25(OH)2D3. No correlation was found between bilirubin levels and either metabolite (Figure 3a,b) (Spearman’s rho = 0.103; *p* = 0.226 (1,25(OH)2D3); 0.007; *p* = 0.938 (25(OH)D3)). We next investigated if female and male patients with elevated bilirubin (characterized by the sex-specific cutoffs) presented differences in either 25(OH)D3 or 1,25(OH)2D3 levels. As observed in Figure 3c,d, no significant differences were found for female patients. The same result was observed for male patients (Figure 3e,f). Independent of an elevated bilirubin level, the levels of both vitamin D3 metabolites remained comparable.

### 2.4. Correlation of eGFR Levels with 25(OH)D3 and 1,25(OH)2D3

**Figure 4:**
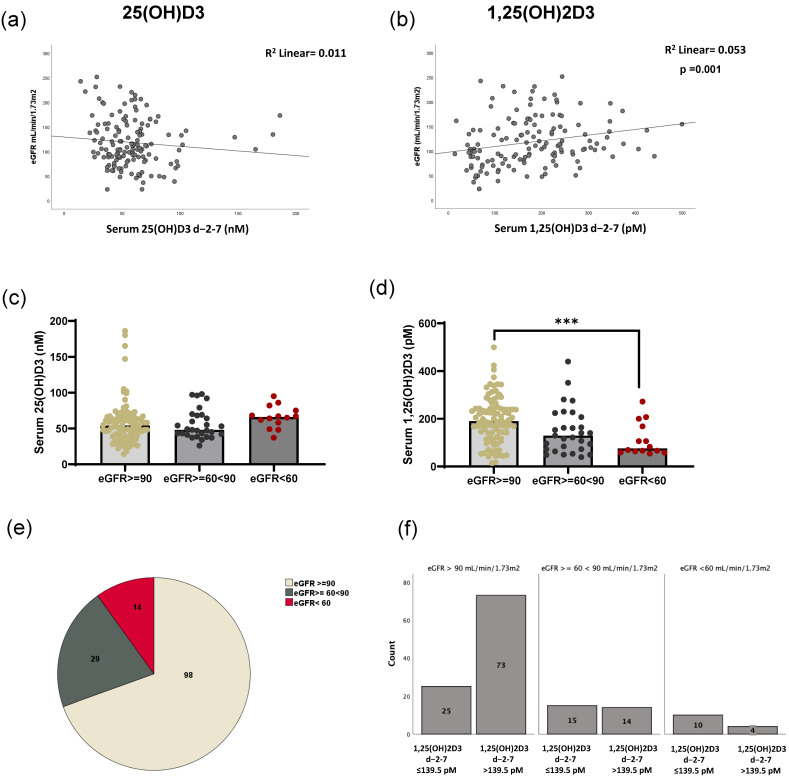
Correlation of eGFR and 25-hydroxyvitamin D3 and 1,25-dihydroxyvitamin D3 serum levels in HSCT patients. The correlation between eGFR values and 25(OH)D3 (**a**) and 1,25(OH)2D3 (**b**) in patient serum at peri-transplant is shown. (**c**) The level of 25(OH)D3 stratified accordingly to eGFR levels. (**d**) The level of 1,25(OH)2D3. (*** *p* ≤ 0.001; Bonferroni test). (**e**) The patient distribution according to eGFR values. (**f**) The number of patients below or above the 1,25(OH)2D3 cutoff of 139.5 pM, accordingly to eGFR stratification.

As HSCT patients frequently experience kidney injury, we investigated whether patients with any form of kidney damage exhibited differences in their ability to metabolize vitamin D3. As shown in Figure 4a, no correlation was found between eGFR and peri-transplant 25(OH)D3 levels. However, a positive correlation was found between eGFR and peri-transplant 1,25(OH)2D3 serum levels (Spearman r = 0.29; *p* < 0.01). Patients with higher serum 1,25(OH)2D3 had higher eGFRs. In Figure 4c,d, the patients are divided into eGFR ≥ 90 (*n* = 98), 60–90, (*n* = 29), or <60 mL/min/1.73 m^2^ (*n* = 14), indicative of normal, slightly impaired, or impaired kidney function, respectively, and the serum levels of 25(OH)D3 and 1,25(OH)2D3 were evaluated. No differences in 25(OH)D3 levels by eGFR category were found (Figure 4c). Conversely, 1,25(OH)2D3 levels gradually decreased in patients with increased kidney function impairment (Figure 4d). A statistical difference was observed in the levels of 1,25(OH)2D3 between patients with eGFRs above 90 (normal) and patients with eGFRs below 60 (impaired kidney function). The distribution of eGFRs among our patient cohort is illustrated in Figure 4e, indicating that most patients fell within the normal eGFR range. Figure 4f shows the number of patients above and below the 1,25(OH)2D3 cutoff of 139.5 pM in relation to their eGFR levels. We observed that the majority of patients who met the 139.5 pM 1,25(OH)2D3 cutoff had eGFR values exceeding 90 mL/min/1.73 m^2^. However, some patients with lower eGFRs were also able to achieve the optimal cutoff.

### 2.5. Clinical Relevance for 1,25(OH)2D3 in Predicting TRM in Patients Stratified According to Their eGFR Values

As we observed a positive correlation between 1,25(OH)2D3 and eGFRs, we next examined if 1,25(OH)2D3 levels still predicted the survival of the patients accounting for their kidney function.

When applying Cox proportional hazards models adjusted for baseline risk factors including eGFRs at peri-transplant (Table 1), we found statistically significant association for high 1,25(OH)2D3 serum levels and lower one-year TRM (HR = 0.99; *p* = 0.001) that remained significant upon adjustment for several risk factors, including eGFR levels (HR = 0.99; *p* = 0.004).

Afterwards, we divided the patients according to their calculated eGFRs and looked at one-year survival applying the 139.5 pM 1,25(OH)2D3 cutoff.

**Figure 5:**
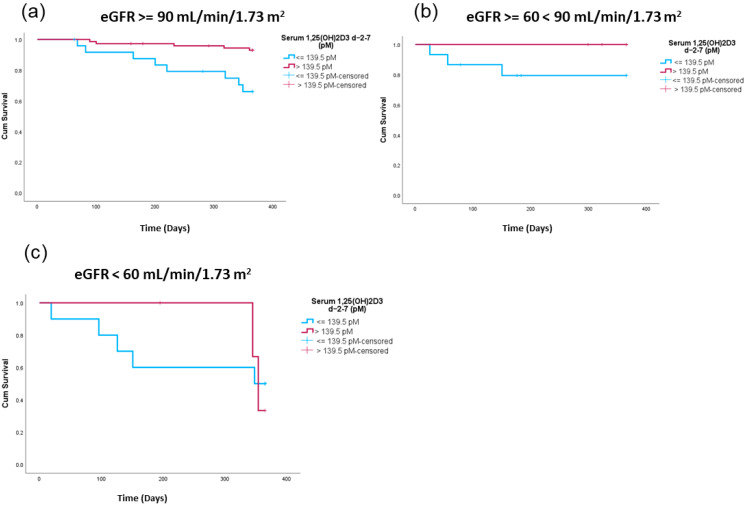
Treatment-related survival comparing patients with high versus low peri-transplant 1,25(OH)2D3 serum levels in relation to eGFR levels. Shown are Kaplan–Meier curves comparing patients with high peri-transplant 1,25(OH)2D3 levels (red) versus patients with lower peri-transplant 1,25(OH)2D3 levels (blue) for patients with eGFR levels > 90 mL/min/1.73 m^2^ (log-rank test *p* = 0.001) (**a**); ≥60 mL/min/1.73 m^2^ < 90 mL/min/1.73 m^2^ (log-rank test *p* = 0.079) (**b**) and eGFR < 60 mL/min/1.73 m^2^ (log-rank test *p* = 0.896) (**c**).

As observed in Figure 5a–c, high 1,25(OH)2D3 peri-transplant levels were related to lower one-year TRM in all eGFR categories, as we observed clearly separated Kaplan–Meier survival curves in all of the eGFR strata. However, due to the nature of the data distribution, only eGFRs above 90 were significantly different. (eGFR > 90 log-rank test *p* = 0.001; eGFR > 60 < 90 log-rank test *p* = 0.079; eGFR < 60 log-rank test *p* = 0.896).

## 3. Discussion

The impact of vitamin D status on overall health is widely recognized. Still, a full consensus on an adequate 25(OH)D3 level does not exist. However, it is commonly agreed that serum 25(OH)D3 below 30 nmol/L is associated with vitamin D deficiency and values above 50 nmol/L are considered adequate [25]. A number of studies have described 25(OH)D3 insufficiency after HSCT [10,26,27,28,29,30,31,32]. However, the role of the active 1,25(OH)2D3 metabolite has not been fully examined. We previously demonstrated that early 1,25(OH)2D3 levels are predictive for one-year transplant-related mortality after HSCT, and were the first to analyze the impact of the active vitamin D3 metabolite in the peri-transplant setting [12].

Several factors may contribute to vitamin D3 deficiency after transplantation, including decreased vitamin D3 synthesis by the skin due by lack of sun exposure, diminished nutrient absorption, or altered vitamin D metabolism due to kidney and liver injury [33].

The importance of vitamin D in the context of HSCT can be attributed to the ability of vitamin D3 to support a tolerogenic environment, promoting Th2 and Treg responses, to foster a healthy microbiome, and to be involved in hematopoiesis [34,35].

In the present study, the patients were supplemented with high-dose vitamin D3 and the median peri-transplant level of 25(OH)D3 was 54 nM, a value considered to be adequate. As published by Peter et al. [12], no relationship between 25(OH)D3 levels at this time interval and TRM was found. However, a significance for 1,25(OH)2D3 levels was observed.

As the primary pathway for vitamin D metabolism involves the liver and kidneys—both of which are targets in GvHD—we investigated whether changes in clinical parameters used to assess the overall function of these organs were correlated with 25(OH)D3 and 1,25(OH)2D3 levels. The main reason behind this study was to determine whether our observations indicated a causal relationship between low 1,25(OH)2D3 levels and transplantation outcomes, or if low 1,25(OH)2D3 levels merely reflected a poorer health status among the patients. In our previous publication, we adjusted for the Karnofsky performance score as an indicator of the overall health condition of our patients; however, this adjustment did not alter the statistically significant association between 1,25(OH)2D3 levels and TRM [12]. This indicates that, in principle, the overall health status of the patients cannot explain the variations in 1,25(OH)2D3 levels. We also investigated the relationship between vitamin D3 levels and patient sex, as several factors related to liver and kidney function are sex-dependent. Additionally, numerous studies have reported differences in vitamin D3 metabolism between women and men [36].

We observed that during the peri-transplant period, levels of 1,25(OH)2D3 significantly increased in both female and male patients. However, 1,25(OH)2D3 levels were notably higher in female patients compared with their male counterparts, while no sex-dependent differences in 25(OH)D3 levels were observed. It can be speculated that the increase in 1,25(OH)2D3 levels was related to the conditioning regimens, as we have previously shown that ATG can modulate CYP27B1 expression, leading to enhanced production of 1,25(OH)2D3 [37]. Whether female patients are more susceptible to increased 1,25(OH)2D3 production, possibly through a hormone-dependent mechanism, warrants further investigation. Pasing et al. studied transcriptome changes upon vitamin D3 supplementation and although 25(OH)D3 levels did not differ between women and men, the investigators found that vitamin D3 regulated 3.2 times more genes in women compared with men [38]. As there is a lack of studies measuring the active form 1,25(OH)2D3, no assumption can be made as to whether our data were corroborated by others. However, since sex-dependent differences in 1,25(OH)2D3 production could only be observed during the peri-transplant window, distinct hormonal patterns between men and women might impact sensitivity to conditioning regimens. Importantly, for both female and males, the 1y-TRM correlated with lower 1,25(OH)2D3 levels.

As the first hydroxylation step of vitamin D3 metabolism occurs in the liver with the production of 25(OH)D3, we first analyzed the levels of bilirubin and CRP in both male and female patients and its association with vitamin D3 levels. As expected, men had higher bilirubin levels compared with women, but no correlation between liver by-products and vitamin D3 metabolites was found. Also, when dividing the patients according to bilirubin levels, we observed no significant differences in vitamin D3 levels between patients. This indicates that the differences observed at peri-transplant can probably not be attributed to liver function.

The second vitamin D3 hydroxylation step mainly occurs in the kidney. Here, the active metabolite 1,25(OH)2D3 is synthetized. eGFR is a parameter normally used to attest for kidney function. It can be calculated based on serum creatinine or cystatin C levels. Although creatinine is more commonly used in a clinical setting, its application has certain limitations, particularly in patients with lower muscle mass. Cystatin C is considered a superior filtration marker for calculating estimated glomerular filtration rate (eGFR) since it is not tubularly secreted; however, its use is often restricted due to higher costs and the fact that it can be influenced by various conditions, such as thyroid disease, adiposity, and underlying inflammation [20,39]. As this study was retrospective, we could only use creatinine levels to assess kidney function. Although our assessment of kidney function was somewhat simplistic, it enabled us to show a positive correlation between eGFRs and high peri-transplant levels of 1,25(OH)2D3 that suggested that patients with impaired kidney function are at higher risk for 1y-TRM. When we stratified our patients according to their eGFRs we could observe that the vast majority (69%) had normal eGFR values, and even when analyzing patients either with early- or advanced-stage kidney disease, 1,25(OH)2D3 levels were still predictive for 1y-TRM. In line with this, in a multivariate analysis we found that 1,25(OH)2D3 levels remained significant for predicting 1-year TRM even if eGFRs were considered. This indicates that high 1,25(OH)2D3 levels seem to foster an environment where the patients are at lower risk of 1y-TRM—which is what was found for all eGFR groups. However, several influencing factors have to be taken into consideration. We used the broadly used and clinically accepted model of blood creatinine for estimation of kidney function. As already mentioned, creatinine, as a parameter for eGFR calculation, displays several limitations. In terms of this study, it has to be emphasized that eGFR, a parameter used to assess the renal filtration rate, is probably associated with, but does exactly represent, the kidneys’ endocrine function. Thus, further studies assessing the correlation between the activity of the enzyme system responsible for hydroxylating 25(OH)D3 to 1,25(OH)2D3, and 1y-TRM are needed. It is also known that vitamin D3 metabolism is tightly regulated mainly by PTH, FGF23, and 1,25(OH)2D3. When calcium and phosphate levels are low, PTH rises and stimulates CYP27B1 transcription, resulting in 1,25(OH)2D3 production. The latter will enhance FGF23 production in bones, which will consequently diminish CYP27B1 and increase CYP24A1 activity. Therefore, PTH increases CYP27B1 expression, while FGF23 and 1,25(OH)2D3 suppress it. Our data revealed no significant changes regarding phosphate levels at peri-transplant. However, when looking at the calcium levels, one can observe a trend for diminished calcium levels at days −2 to 7. At the same time, 1,25(OH)2D3 levels increased. One can also speculate that the observed 1,25(OH)2D3 peak was a response to lower calcium levels.

Furthermore, it is known that immune cells, such as macrophages and dendritic cells (DCs), express CYP27B1 and are capable of 1,25(OH)2D3 production [40,41,42]. Thus, extra-renal production of 1,25(OH)2D3 must be taken into consideration, too. If the observed peak in 1,25(OH)2D3 cannot be explained by liver or kidney function differences, one can speculate that differences in macrophage/DC populations could be observed in those patients with higher 1,25(OH)2D3 levels. Thus, further studies are needed to evaluate and correlate vitamin D3 levels and immune-cell composition of the patients.

Additionally, it is known that vitamin D3 has an impact on the microbiome [43,44,45] and that the latter is a huge player in the context of HSCT [46,47,48,49]. Studies demonstrate that GvHD patients often suffer from dysbiosis, mainly manifested by decreased microbial diversity, shifts in gut microbial composition, and microbiome-derived metabolites.

Currently it is also speculated that the microbiome could influence vitamin D3 levels, as some bacterial species are capable of vitamin D3 hydroxylation [50]. Having this interplay in mind, it would be useful to investigate and correlate serum vitamin D3 levels (25(OH)D3 and 1,25(OH)2D3) with bacterial species composition and transplantation outcome.

Patients with higher 1,25(OH)2D3 levels may have a microbiome that fosters better gut health, exemplified by higher bacterial diversity and balanced microbial metabolites such as short-chain fatty acids (SCFAs), bile acids (BAs) and indoles [51]. In addition, the benefit of vitamin D3 for tight junction expression and gut barrier integrity is also recognized [52].

The action of 1,25(OH)2D3 is mediated through the vitamin D receptor (VDR). We also evaluated the importance of VDR expression in the HSCT context [53]. We previously demonstrated that patients suffering from severe acute gastrointestinal GvHD show significant downregulation of the VDR compared with patients with mild or no GvHD, and that low VDR expression was an independent risk for TRM [53]. These data further highlight the importance of an adequate vitamin D3 metabolism for transplantation outcome that does not seem to be merely indicative for the overall health condition of the patient.

Taken together, our results suggest that high peri-transplant 1,25(OH)2D3 levels, which have protective effects on one-year survival following HSCT, positively correlate with kidney function as estimated by blood creatinine levels. Although female patients exhibited higher 1,25(OH)2D3 levels at the time of transplantation compared with male patients, sex was not a significant variable influencing transplantation outcomes. Furthermore, kidney function alone could not account for why some patients failed to reach the optimal 1,25(OH)2D3 threshold. The precise mechanism by which 1,25(OH)2D3 functions as a protective metabolite warrants further investigation in a future prospective study.

## 4. Materials and Methods

### 4.1. Patient Characteristics

A total of 141 patients participated in this study. The cohort consisted of patients undergoing hematopoietic stem-cell transplantation (HSCT) at the Regensburg University Medical Center between May 2012 and February 2015 (Table S1). All HSCT patients received oral high-dose vitamin D3 supplementation (Vigantol oil, 20,000 IU/mL, Merck, Darmstadt, Germany), which included an initial dose of 50,000 IU upon hospital admission (days −16 to −6), followed by daily administration of 10,000 IU. To monitor serum levels of 25-hydroxyvitamin D3 and 1,25-dihydroxyvitamin D3, blood samples were collected repeatedly during the inpatient stay and subsequently during routine outpatient visits. Measurements were conducted at least once during the specified time intervals. In cases where multiple measurements were available for the same interval, the median value was utilized. Serum calcium levels were assessed twice a week. The described supplementation regimen was maintained until patients achieved serum levels of 150–200 nmol/L, with subsequent dose adjustments made to prevent levels exceeding 150–200 nmol/L. The cohort analyzed in the present study has been described in detail in [10].

### 4.2. Vitamin D Measurement

Vitamin D levels were measured immediately after serum withdrawal or from sera stored at −80 °C by the Department of Clinical Chemistry at the University Medical Center of Regensburg, Germany. From May 2012 to October 2014, 25-hydroxyvitamin D3 serum levels were analyzed using a chemiluminescence immunoassay, following the manufacturer’s instructions (Immunodiagnostic Systems, Frankfurt am Main, Germany). After confirming comparability, from November 2014 onward, 25-hydroxyvitamin D3 serum levels were analyzed using liquid chromatography high-resolution tandem mass spectrometry, as described elsewhere [54]. Additionally, 1,25-dihydroxyvitamin D3 levels were measured using a radioimmunoassay, in accordance with the manufacturer’s instructions (Immunodiagnostic Systems, Frankfurt am Main, Germany), by the Department of Clinical Chemistry at the University Medical Center of Regensburg, Germany.

### 4.3. Sample Processing and Laboratory Analysis

Blood samples were collected upon hospital admission and subsequently during the inpatient stay, as well as during routine outpatient visits. Routine clinical chemistry markers, including bilirubin, creatinine, lactate dehydrogenase (LDH), and C-reactive protein (CRP), were measured using a cobas c 503 analytical unit from Roche Diagnostics Ltd. (Rotkreuz, Switzerland) at the Institute of Clinical Chemistry and Laboratory Medicine of the University Hospital Regensburg.

### 4.4. eGFR Calculation

The creatinine-based eGFR was calculated using the CKD-EPI 2021 equation: eGFR = 142 × min(standardized Scr/K,1) α × max(standardized Scr/K,1) −1.200 × 0.9938 age in years × 1.012 [if female], where

Scr = serum creatinine in mg/dL; K = 0.7 (females) or 0.9 (males); α = −0.241 (females) or −0.302 (males); min(standardized Scr/K,1) = the minimum of Scr/K or 1; max(standardized Scr/K,1) = the maximum of Scr/K or 1.

### 4.5. Statistical Analysis

Statistics were calculated using SPSS Statistics version 26 (IBM, Armonk, NY, USA) and/or GraphPad Prism (v10, GraphPad Software, La Jolla, CA, USA). Comparisons between groups were performed using the appropriate statistical methods depending on Gaussian distributions, number of groups, and variables. A value of *p* < 0.05 was considered statistically significant. To examine one-year survival, a Kaplan–Meier curve was generated to visualize differences between patients above or below the defined 1,25(OH)2D3 cutoff in relation to eGFR values (above 90 mL/min/1.73 m^2^ was considered the normal range, below 89 and above 60 meant slightly impaired kidney function, and below 60 was considered as impaired kidney function). The association with time-to-death was further evaluated using a Cox proportional hazard model unadjusted and adjusted for baseline risk factors (age, sex, ATG prophylaxis, severe GvHD).

## Figures and Tables

**Table 1:** Association of peri-transplant 1,25(OH)2D3 serum levels with TRM. Shown are results from Cox proportional hazard models for the peri-transplant 1,25(OH)2D3 serum levels from days −2 to 7 with TRM within the 1st year after transplantation without and with adjustment for risk factors and sensitivity analysis. *p* values ≤ 0.05 are marked in bold.

Cox Regression—1,25(OH)2D3			
Peri-Transplant (d (−2)–7)			
	#At Risk/TRM	Exp(B)/HR (95% CI)	*p* Value
**Unadjusted**	141/23		
Serum 1,25(OH)2D3		0.991 (0.986; 0.997)	**0.001**
**Adjusted I**	141/23		
Serum 1,25(OH)2D3		0.992 (0.987; 0.997)	**0.004**
Male sex		1.417 (0.605; 3.319)	0.422
Age		1.076 (1.017; 1.138)	**0.010**
**Adjusted II**	141/23		
Serum 1,25(OH)2D3		0.991 (0.985; 0.997)	**0.003**
Male sex		0.494 (0.181; 1.350)	0.169
Age		1.048 (0.991; 1.107)	0.100
aGvHD (yes/no)		16.401 (6.176; 43.554)	**<0.0005**
ATG (yes/no)		1.092 (0.401; 2.974)	0.863
**Adjusted III**	141/23		
Serum 1,25(OH)2D3		0.991 (0.986; 0.997)	**0.004**
Male sex		0.508 (0.187; 1.383)	0.173
Age		1.045 (0.987; 1.106)	0.116
aGvHD (yes/no)		15.573 (5.810; 41.743)	**<0.0005**
ATG (yes/no)		1.055 (0.388; 2.865)	0.916
eGFR		0.996 (0.986; 1.007)	0.508

## Data Availability

The original contributions presented in the study are included in the article/Supplementary Materials; further inquiries can be directed to the corresponding author.

## Supplementary Materials

The following supporting information can be downloaded at: https://www.mdpi.com/article/10.3390/ijms26072866/s1.
