# Supplementary material for: Impact of Liver and Kidney Function on Vitamin D3 Metabolism in Female and Male Patients Undergoing Allogeneic Hematopoietic Stem-Cell Transplantation
Source: Int J Mol Sci. 2025 Mar 21;26(7):2866. doi: 10.3390/ijms26072866 (PMC11988875; doi:10.3390/ijms26072866)
Supplement: Supplementary file 1 [file ijms-26-02866-s001.zip › ijms-3442973-supplementary.pdf]

Figure S1 – Time trend of Calcium levels in relation to 25-Hydroxyvitamin D3 and 1,25-Dihydroxyvitamin D3 levels in female and male patients

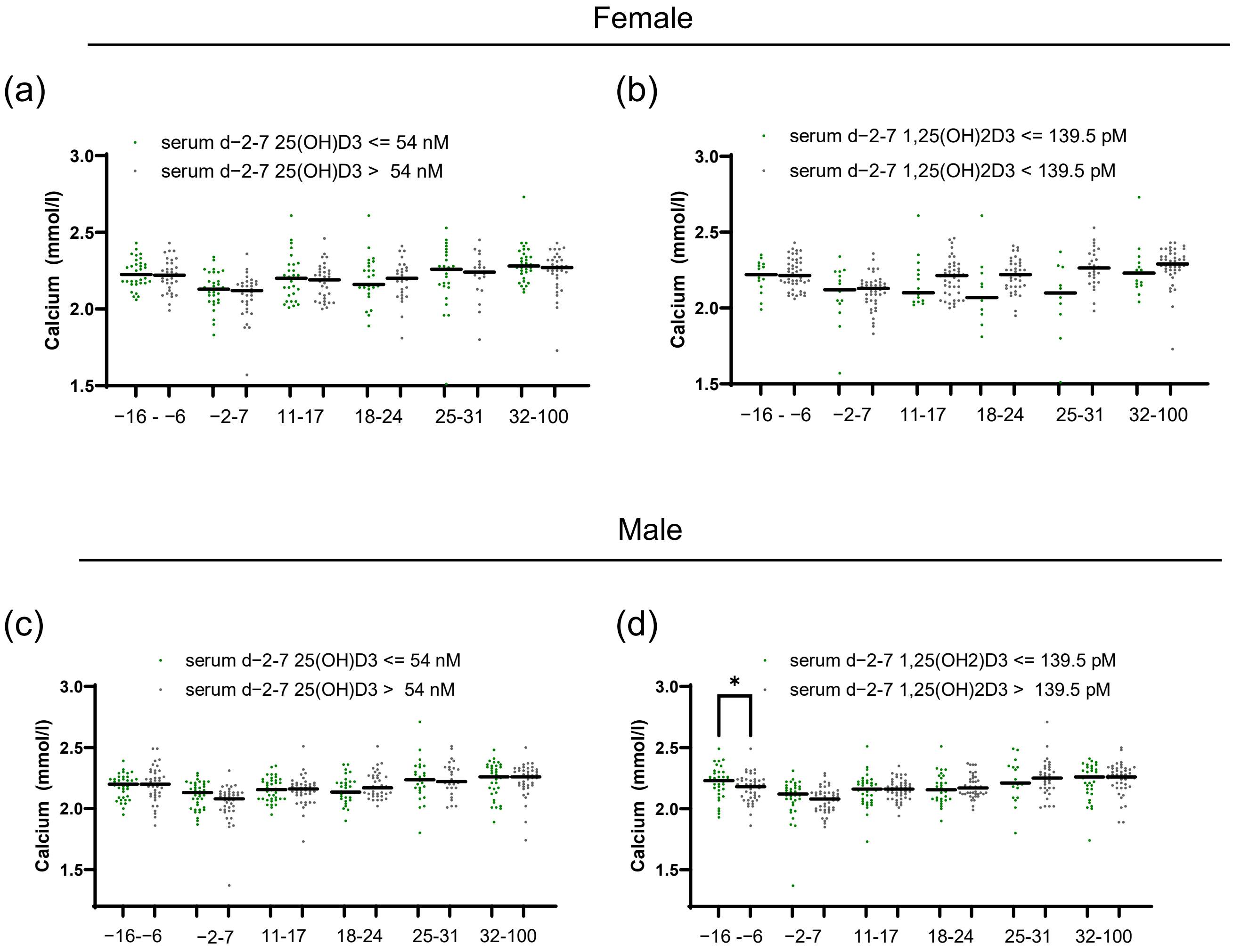

Figure S1 – Time trend of Calcium levels in relation to 25-Hydroxyvitamin D3 and 1,25-Dihydroxyvitamin D3 levels in female and male patients. In a) the calcium levels for female patients are depicted during the indicated time intervals in relation to serum 25(OH)D3 levels (above or below 54 nM). Panel b) depicts calcium in female patients above/below the 139,5 pM 1,25(OH)2D3 cut-off. In panel c) the serum calcium values are depicted above or below 54 nM for male patients and panel d) shows the calcium values for male patients above or below the 139.5 pM cut-off. Statistical analysis was performed using Mann-Whitney U test. (*P ≤ 0.05).

Figure S2 – Time trend of Phosphate levels in relation to 25-Hydroxyvitamin D3 and 1,25-Dihydroxyvitamin D3 levels in female and male patients

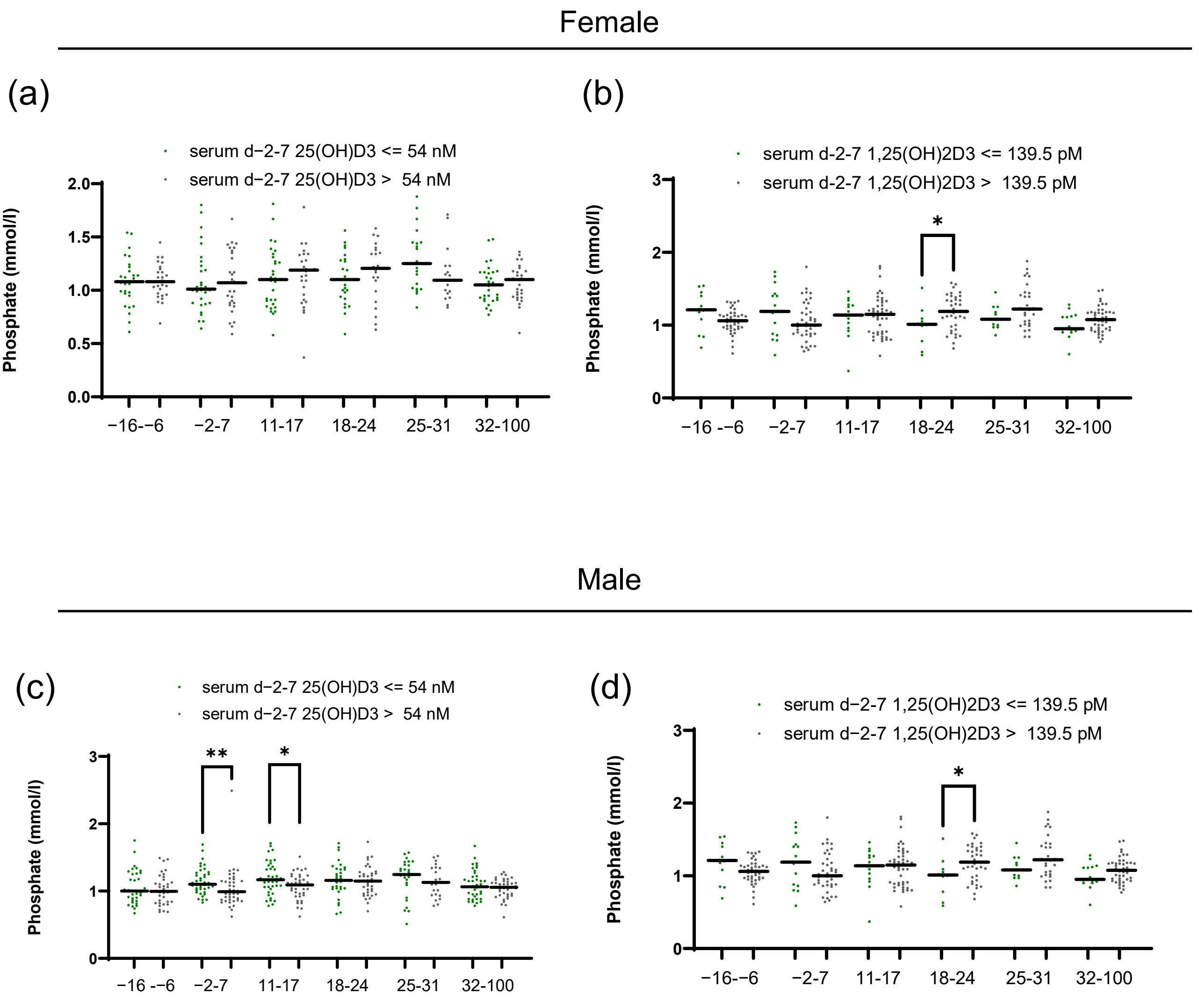

Figure S2 – Time trend of Phosphate levels in relation to 25-Hydroxyvitamin D3 and 1,25-Dihydroxyvitamin D3 levels in female and male patients. In a) the phosphate levels for female patients are depicted during the indicated time intervals in relation to serum 25(OH)D3 levels (above or below 54 nM). Panel b) depicts phosphate in female patients above/below the 139.5 pM 1,25(OH)2D3 cut-off. In panel c) the serum phosphate values are depicted above or below 54 nM for male patients and panel d) shows the phosphate values for male patients above or below the 139.5 pM cut-off. Statistical analysis was performed using Mann-Whitney U test. (*P ≤ 0.05; **P ≤ 0.01).

Figure S3 – Time trend and correlation of **CRP levels** with 1,25-Dihydroxyvitamin D3 in female and male patients

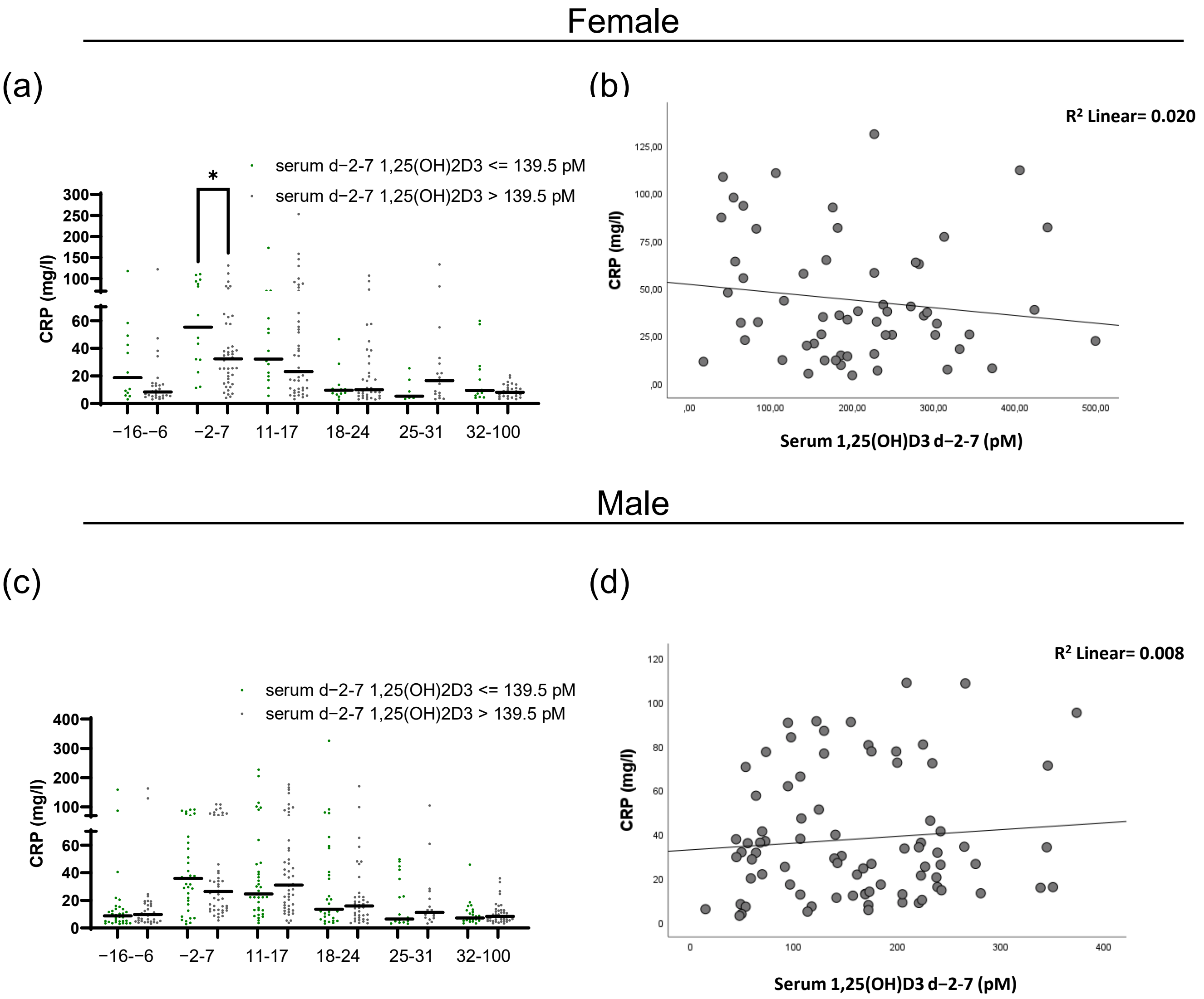

Figure S3 – Time trend and correlation of CRP levels with 1,25-Dihydroxyvitamin D3 in female and male patients. In a) CRP levels are shown in the indicated time intervals for female patients below (blue) or above (red) the 1,25(OH)2D3 cut-off of 139.5 pM. The correlation between CRP and 1,25(OH)2D3 in serum in the peri-transplant interval (days −2-7) are shown in b). C) depicts CRP levels in the indicated time intervals for male patients below (blue) or above (red) the 1,25(OH)2D3 cut-off of 139.5 pM and d) shows the correlation between CRP levels and 1,25(OH)2D3 levels for male patients at peri-transplant (days −2-7). Statistical analysis was performed using Mann-Whitney U test. (*P ≤ 0.05)

Figure S4 – Time trend and correlation of **LDH levels** with 1,25-Dihydroxyvitamin D3 in female and male patients

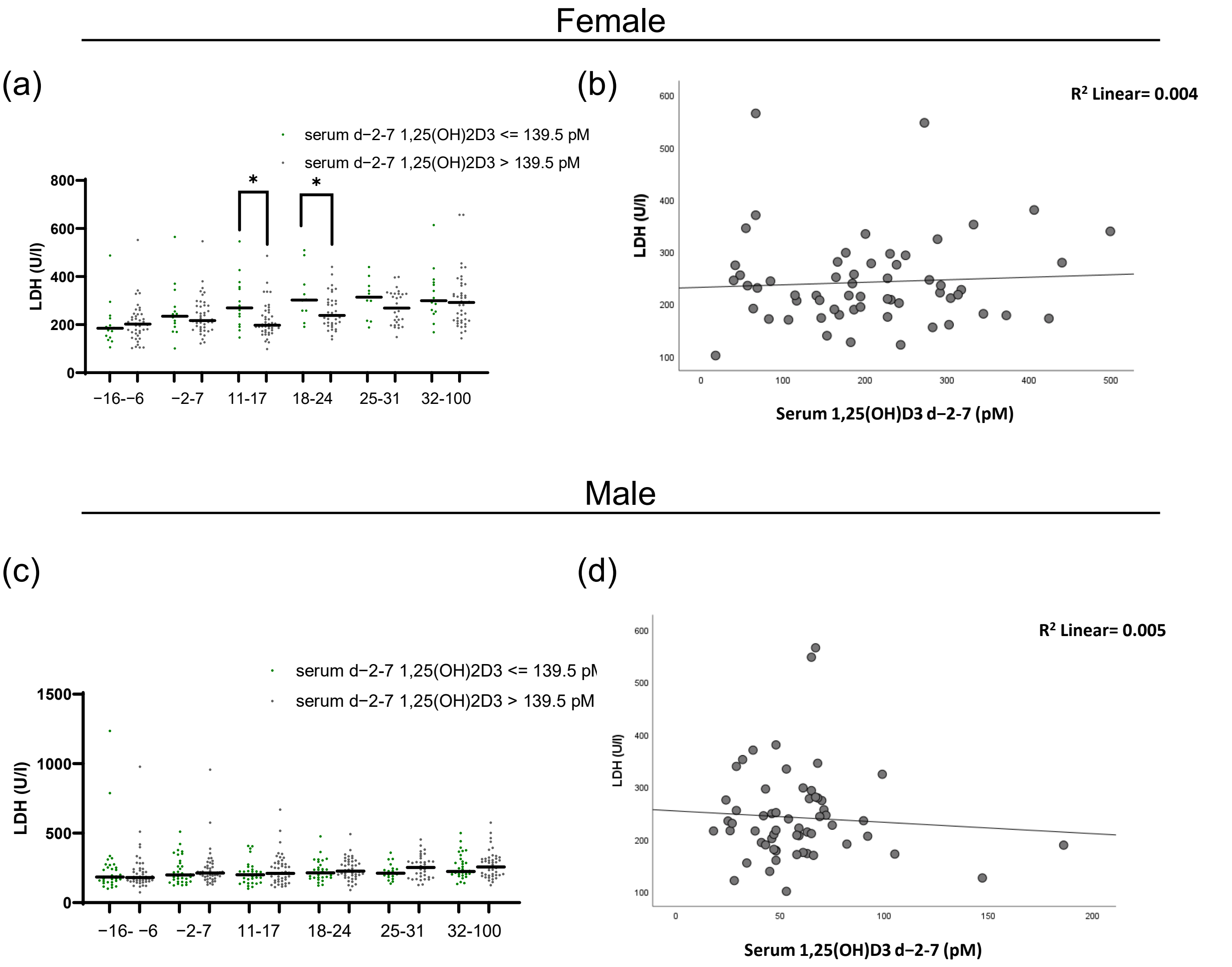

Figure S4 – **Time trend and correlation of LDH levels with 1,25-Dihydroxyvitamin D3 in female and male patients.** In a) LDH levels are shown in the indicated time intervals for female patients below (blue) or above (red) the 1,25(OH)2D3 cut-off of 139.5 pM. The correlation between LDH and 1,25(OH)2D3 in serum in the peri-transplant interval (days −2-7) are shown in b). C) depicts LDH levels in the indicated time intervals for male patients below (blue) or above (red) the 1,25(OH)2D3 cut-off of 139.5 pM and d) shows the correlation between LDH levels and 1,25(OH)2D3 levels for male patients at peri-transplant (days −2-7). Statistical analysis was performed using Mann-Whitney U test. (*P ≤ 0.05; **P ≤ 0.01).

Table S1. Patient characteristics at baseline

**characteristic**	**discovery (n=141)**
study center	Regensburg
vitamin D3 suppl.*	high-dose
male sex	82 (58,2%)
median age (range)[yr]	56 (26-70)
diagnosis	
aplastic anemia	2 (1.4%)
acute leukemia	75 (53.2%)
Morbus Hodgkin	4 (2.8%)
MDS	17 (12.1%)
MPN	4 (2.8%)
NHL	34 (24.1%)
PMF	5 (3.5%)
late tumor stage†	75 (58.6%)
unrelated donor	100 (70.9%)
ATG before HSCT yes	100 (70.9%)
standard conditioning‡	13 (9.2%)
steroids ≥1 mg/kg§	65 (53.3%)
Karnofsky score <90¶	46 (32.6%)

suppl.=supplementation, yr=years, MDS=myelodysplastic syndrome, MPN=myeloproliferative neoplasm, NHL=Non-Hodgkin lymphoma, PMF=primary myelofibrosis, HSCT=hematopoietic stem cell transplantation, ATG=antithymocyte globulin

*high dose supplementation (50.000 IU p.o. at start, followed by 10.000 IU/d p.o.) was adjusted for patients with 25-hydroxyvitamin-D3 levels higher than 200 nmol/L

†at enrollment (before HSCT), no tumor stage grading available for 15 patients, classification according to EBMT risk score47

‡missing information on conditioning from 5 patients

§missing information on steroid treatment 21 patients

¶missing information on Karnofsky performance score from 5 patients
